# Focusing on Alzheimer’s Disease: Data Comparison and Policy Implications Between China and Four Provinces

**DOI:** 10.1093/geroni/igaf122.2853

**Published:** 2025-12-31

**Authors:** Wangyizhuang Lyu, Hong Mi

**Affiliations:** Zhejiang University, Hangzhou, Zhejiang, China (People’s Republic); Zhejiang University, Hangzhou, Zhejiang, China (People’s Republic)

## Abstract

By 2050, China’s population aged 65 and above is projected to exceed 400 million, with Alzheimer’s disease (AD) prevalence rising sharply with age. Diagnosis and treatment remain underdeveloped, leading to underdiagnosis in early stages and significant urban-rural disparities in care resources. These gaps may widen as demographic aging accelerates, while long-term care costs pose a heavy burden on families and society, requiring early policy planning. This study, using data from the Fifth Sampling Survey on the Living Conditions of Urban and Rural Older Adults, analyzes AD prevalence across China and four provinces—Shandong, Zhejiang, Sichuan, and Heilongjiang—revealing key findings: (1) AD affects 1.58% of individuals aged 60 and above, with higher rates in rural areas (1.86%) than urban areas (1.28%). Dementia is slightly more common among urban males and rural females. (2) Prevalence rises notably after 70, peaking at 70–75 for men and after 70 for women, with men developing dementia 5–10 years earlier. (3) Regional differences exist: Zhejiang’s peak occurs after 75, with women affected earlier; in Shandong, female prevalence rises faster post-75; Sichuan’s peak is at 75–85; Heilongjiang shows a gender-based timing gap. (4) Rural disability rates in Zhejiang and Heilongjiang are below the national average, likely due to higher urbanization. (5) China’s higher dementia rates and lower life expectancy, compared to global averages, highlight the urgent need to incorporate long-term dementia care into the social security system.

